# Differential IgM expression distinguishes two types of pediatric Burkitt lymphoma in mouse and human

**DOI:** 10.18632/oncotarget.11531

**Published:** 2016-08-23

**Authors:** Anthony B. Eason, Sang-Hoon Sin, Carolina Lin, Blossom Damania, Steven Park, Yuri Fedoriw, Carlos E. Bacchi, Dirk P. Dittmer

**Affiliations:** ^1^ Lineberger Comprehensive Cancer Center, the University of North Carolina at Chapel Hill School of Medicine, Chapel Hill, NC, USA; ^2^ Department of Microbiology and Immunology, the University of North Carolina at Chapel Hill School of Medicine, Chapel Hill, NC, USA; ^3^ Department of Medicine, the University of North Carolina at Chapel Hill School of Medicine, Chapel Hill, NC, USA; ^4^ Department of Pathology and Laboratory Medicine, the University of North Carolina at Chapel Hill School of Medicine, Chapel Hill, NC, USA; ^5^ Pathology Reference Laboratory, Botucatu, São Paulo, Brazil

**Keywords:** Burkitt lymphoma, Epstein-Barr virus, IgM, Myc, ibrutinib

## Abstract

Endemic Burkitt lymphoma (eBL) is primarily a childhood cancer in parts of Africa and Brazil. Classic studies describe eBL as a homogeneous entity based on t(8;14) IgH-Myc translocation and clinical response to cytotoxic therapy. By contrast, sporadic BL (sBL) in Western countries is considered more heterogeneous, and affects both children and adults. It is overrepresented in AIDS patients. Unlike diffuse large B cell lymphoma (DLBCL), molecular subtypes within BL have not been well defined. We find that differential IgM positivity can be used to describe two subtypes of pediatric Burkitt lymphoma both in a high incidence region (Brazil), as well as in a sporadic region (US), suggesting the phenotype is not necessarily geographically isolated. Moreover, we find that IgM positivity also distinguishes between early and late onset BL in the standard Eμ-Myc mouse model of BL. This suggests that the t(8;14) translocation not only can take place before, but also after isotype switch recombination, and that IgM-negative, t(8;14) positive lymphomas in children should nevertheless be considered BL.

## INTRODUCTION

Burkitt lymphoma (BL) is an aggressive Non-Hodgkin B cell lymphoma. Histologically, it exhibits a characteristic “starry-sky” appearance due to macrophages laden with phagocytized apoptotic debris within a background of malignant cells. Endemic BL (eBL) is the most common hematopoietic neoplasia of children in Sub-Saharan Africa. Denis Burkitt first described it in 1957 in Kampala, Uganda, and at the time, he noticed an association with Malaria; later, Epstein and Barr also identified the association with Epstein-Barr Virus (EBV) (reviewed in [1–3]). Endemic BL responds well to single agent cyclophosphamide (Cytoxan) or other relatively simple DNA damaging regimes (doxorubicin, vincristine), presumably because of its extremely rapid proliferation rate as ascertained by a Ki-67 index above 95%. eBL is considered to have a very homogenous immunophenotype with few secondary genomic aberrations [4, 5], perhaps because EBV activates oncogenic signaling pathways, which in EBV-negative BL are activated by driver mutations. There is a paucity of well-controlled clinical and molecular studies in regions that are endemic for BL, such as Sub-Saharan Africa.

Sporadic BL (sBL) is a very different disease in Western countries, and its immunophenotype is much more variable. In the 1990s, a subtype of sBL was recognized in HIV+ patients in the US and Europe (reviewed in [6]). Both forms of sBL in HIV+ and HIV- patients are equally proliferative and aggressive, but much more chemoresistant than eBL. In HIV- patients, sBL is seldom EBV-associated, with the exception of Brazilian cohorts that have an EBV expression intermediate between sBL and eBL [7]. HIV is now epidemic in the same regions in which there is eBL; therefore, it is unclear if the classical histological diagnosis and established treatment options still hold.

There also exists the risk of misdiagnosis of BL and aggressive B-cell lymphoma with intermediate features between BL and diffuse large B cell lymphoma (DLBCL) [8]. Overexpression of the proto-oncogene c-Myc is the key molecular driver of BL. The t(8;14)(q24q32) translocation involving the IgH locus is considered a hallmark of eBL and sBL alike [9], though other translocations, t(2;8) or t(8;22), have been described, as well as cases of Myc activation by somatic mutation [10]. Indeed, Myc activation has been shown to occur in many lymphomas, e.g. those with plasmablastic morphology [11], motivating our search for additional markers to distinguish BL from high-grade DLBCL and to discern sub-types of BL.

BL is considered a B cell tumor of germinal center (GC) or post-germinal center origin with a “sky-high” proliferation rate akin to the staggering rate of centroblast proliferation in the dark zone of the GC. Prior to activation, mature B cells express IgM in a monomeric form on their cell surface, along with IgD. After activation, B cells undergo clonal expansion and class switch recombination (CSR), whereupon IgM is downregulated and IgG isotypes are expressed with a different constant region, while maintaining the same variable region. Clonal expansion takes place in the dark zone of the GC, as does IgG hypermutation and CSR. Subsequently, affinity maturation takes place in centrocytes within the less proliferative, light zone of the GC. Low affinity B cells die by apoptosis, whereas high affinity B cells circle back into the dark zone. We were interested in studying IgM expression in pediatric BL, since it allows for the distinction between BL originating in the dark zone of the GC, i.e. before CSR, and BL originating in light zone of the GC, i.e. after CSR.

Here, we report IgM expression as a novel histological marker that distinguishes two subtypes of pediatric t(8;14) BL. Samples were obtained from patients in a sporadic setting (USA), and a high incidence, sporadic-like setting (Brazil). We chose to focus on pediatric patients because they are most likely to have causative primary translocations compared to adult patients; in addition, future potential comparisons to eBL patients, a primarily pediatric population, will be possible. Notably, IgM expression also distinguished early and late onset lymphomas in the standard Eμ-Myc mouse model of BL [12–14]. This suggests that IgM may be useful as a new biomarker to stratify pediatric BL.

## RESULTS

**Figure 1:**
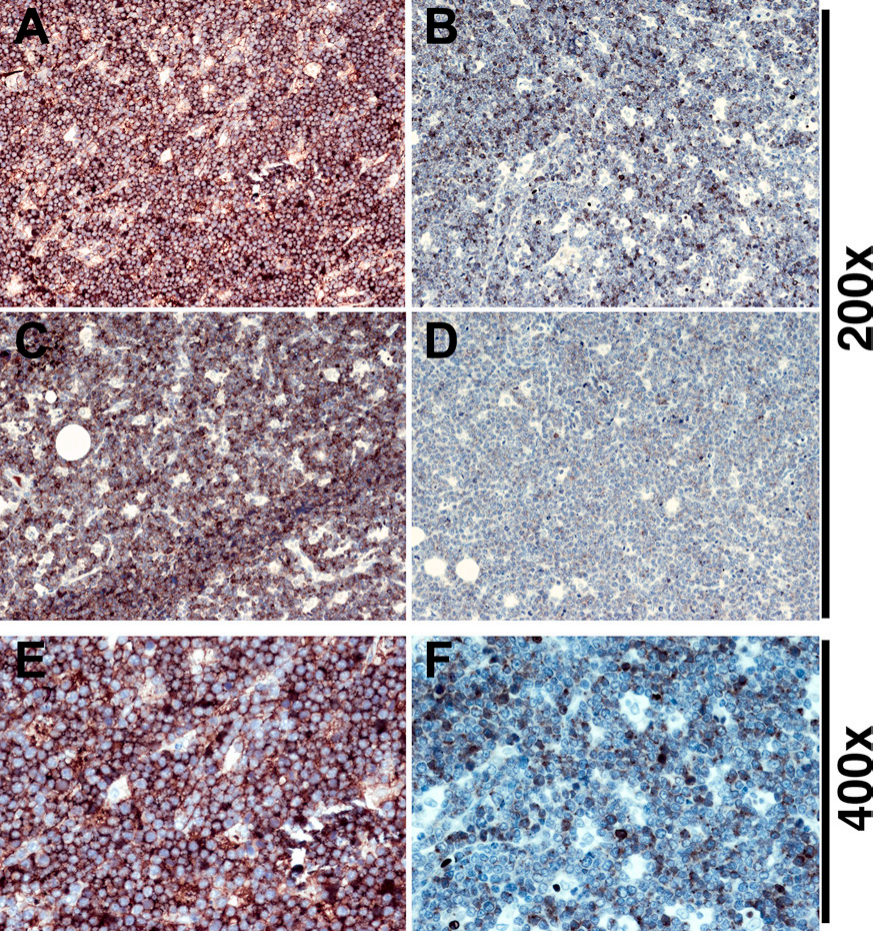
Variant IgM expression in BL (**A**, **B**, **C**, **D**) 200× magnification of different BL cases stained for IgM. (A, C) BL: Dark NovaRED™ staining evident, tumor cells are strongly positive. (B, D) BL: Staining is mostly negative with scant peripheral IgM in weakly positive cells. (**E**) Example of high intensity stain from panel (A) at 400× demonstrates IgM is located at the cell periphery. (**F**) Example of low intensity stain from panel (B) at 400× shows a distinct lack of IgM positive cells in comparison.

First, we evaluated five t(8;14) positive cases of pediatric sBL from the UNC clinic (Table 1). All cases exhibited the prototypical “starry-sky” histopathology, and all cases exhibited nuclear Ki-67 staining in > 95% of tumor cells. As positive control, we used an experimental primary effusion lymphoma (PEL) xenograft, which was equally Ki-67 positive, but did not show the prototypical, interspaced pattern of macrophages. We found sIgM staining to be immensely variable in the BL cases (Figure 1): the cases in panels A and C were IgM positive, whereas the cases in panels B and D were IgM negative. The image in panel E shows a higher magnification of the IgM-positive staining in panel A, and panel F shows a higher magnification of the IgM-weak staining in panel B. Clearly, IgM expression is variable among BL carrying the t(8;14) translocation. On the basis of histology, clinical features (pediatric, neck region) and Myc translocation, it seems highly unlikely that these cases represent misdiagnosed DLBCL or plasmablastic lymphoma (PBL). We interpret this data to establish the existence of two sub-types of sBL: those who are sIgM positive (type A) and those who are sIgM negative (type B).

**Table 1:** Individual UNC cases of pediatric BL

Ki-67^a^	IgM^b^	CD79a	Site	Age	Sex	Myc FISH	Karyotype (NC, not complete)	Stage	BCL-6
3+	3	1	abdominal	11	M	t(8;14)	NC	Abdomen limited, BM neg	NC
3+	1	1	neck	6	M	t(8;14)	46,XY,dup(1)(q?21q?31),?del(6)(q15q21),t(8;14)(q24;q32)[13]/46,XY[7]	IV	NC
3+	2	2	neck	5	M	t(8;14)	NC	Neck limited, BM neg	NC
3+	2	2	neck	5	M	t(8;14)	NC	IV	NC
3+	3	3	pelvic	14	F	t(8;14)	NC	III	Positive

a 3+ refers to > 95% of cells staining with high intensity.

b The numeric scores correspond with the following: 1 = low intensity, 2 = intermediate intensity, 3 = high intensity.

Displayed are relevant immunophenotypical and clinical data on pediatric BL patients at UNC hospital. All patients had > 95% Ki-67 index and t(8;14) translocations, with varying degrees of IgM/CD79a expression and tumor localization.

**Figure 2:**
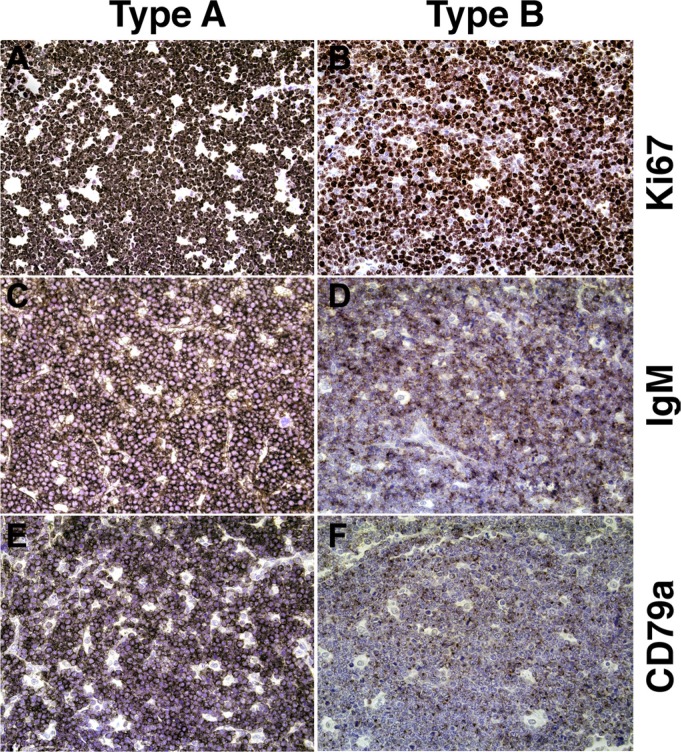
Representative immunophenotypes of type A and type B BL (**A**, **C**, **E**) Sections from a single BL patient classified as “Type A.” (**B**, **D**, **F**) Sections from a second BL patient classified as “Type B.” (A, B) Ki-67: NovaRED staining is dark and prevalent in the nuclei of nearly all cells. (C) IgM staining is dark and concentrated on nearly all cell surfaces, with hematoxylin-counterstained nuclei visible. (D) IgM staining is moderate and scattered, with few cells staining darkly. (E) CD79a staining is dark and concentrated on the most of the cell surfaces (> 85%). (F) CD79a staining is weak, appearing on less than half of cell surfaces. All images at 200× magnification.

CD79a is covalently associated with the B cell receptor; it is also called Ig-α and it is essential for B cell receptor (BCR) signaling and surface expression of IgM [17]. We hypothesized that if sIgM were not expressed, CD79a would be absent as well. This was indeed the case (Figure 2). In IgM-positive, “type A” BL, CD79a staining was readily apparent (Figure 2, panel C, E), whereas in IgM-negative, “type B” BL, CD79a staining was absent in most cells and much reduced in intensity in the remaining IgM faint staining cells (Figure 2, panel D, F). Both cases had equivalent levels of Ki-67 positivity. We concluded that “type B” BL had downregulated BCR expression.

**Figure 3:**
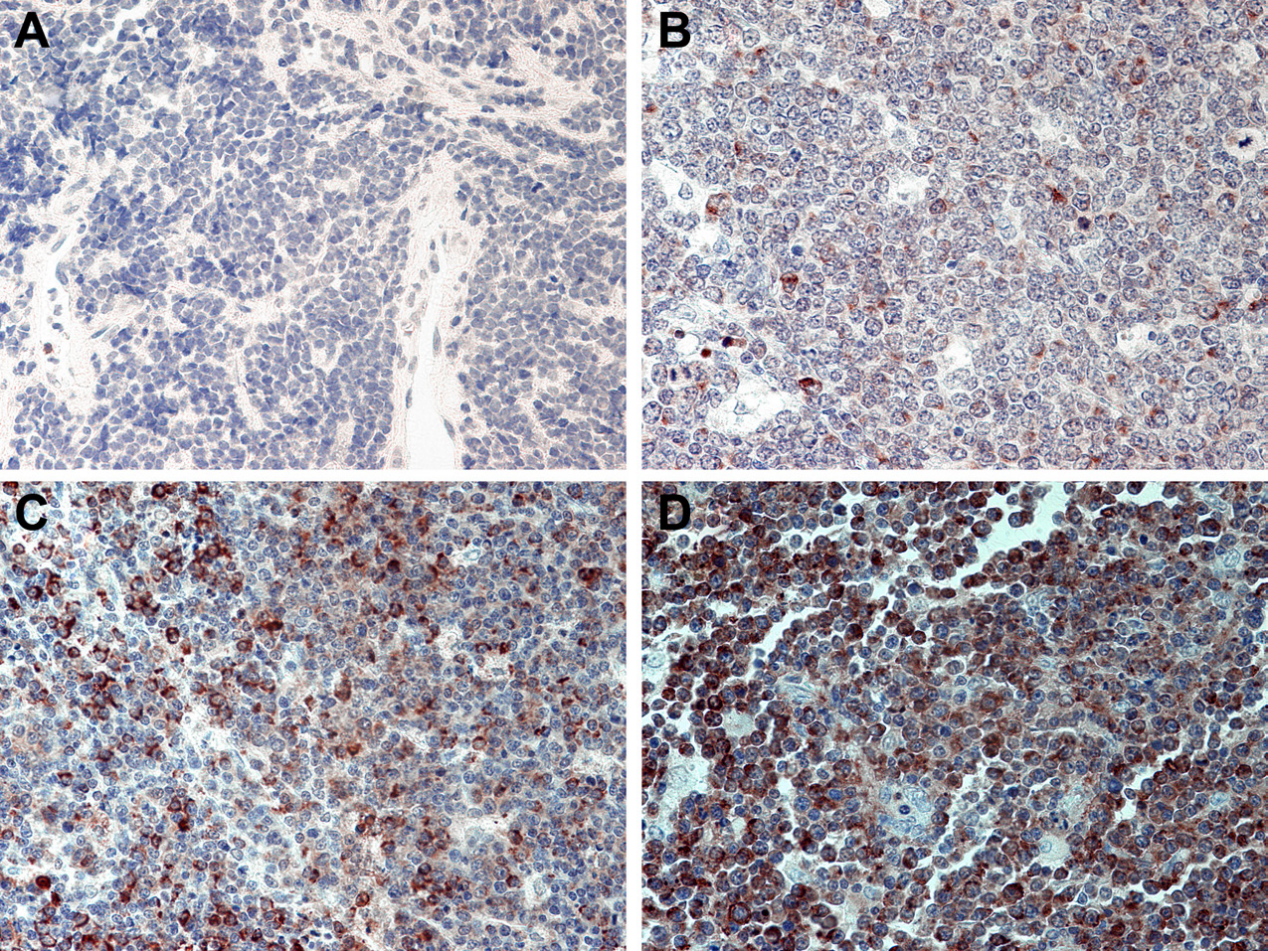
IgM expression on a TMA of Brazilian BL (**A**) Representative tissue section with an intensity score of “0,” representing a negative stain. Hematoxylin counterstain in cell nuclei is visible. (**B**) Representative tissue section with an intensity score of “1,” representing weakly stained tissue. Isolated cells stain weakly with NovaRED. (**C**) Representative tissue section with an intensity score of “2,” representing intermediate intensity. There is a large population of heterogeneously stained cells. (**D**) Representative tissue section with an intensity score of “3,” representing heavily stained tissue. Most of the cells have dark staining that obscures the nucleus significantly. All images at 400× magnification.

Second, we used a tissue micro array (TMA) of cases of sBL from Brazil to independently confirm the presence of these two subtypes. The characteristics of the TMA were previously described [7]. The incidence and composition of BL in the Brazilian general population is primarily of the sporadic-type with a high proportion of pediatric cases and EBV prevalence intermediate between that of endemic and sporadic. All cases were studied by FISH for Myc translocation, and we selected only those sBL cases with unequivocal evidence of Myc translocation, i.e. FISH-MYC index ≥10% [16]. All cases were CD10^pos^, BCL6^pos^, PAX5^pos^, and exhibited a Ki-67 index > 95%. There were 13 total; 12 were from male and 1 from female patients. The median age was 23 (^95%^CI: 15.7 … 34.6). We stringently defined “positive” as a consensus score of “3,” the highest score. We defined “negative” as a consensus score of “1,” the lowest possible score considering we had no patient that scored “0.” We encountered 6 sIgM^pos^ (46%), and 4 sIgM^neg^ (30%) cases in this otherwise homogeneous collection (Figure 3). Three additional cases were homogenously sIgM^pos^ but at lower levels. We conclude that also in this larger collection of sBL, the typical BL cases can be sub-divided into two types: “type A” with high level sIgM expression and “type B” with low level or no sIgM expression. Although we chose a 10% FISH-MYC index threshold in order to filter the results in the most clinically stringent manner possible, we realize it may be acceptable to use a threshold of 2.19% as previously validated [7]. In doing so, the median age is reduced to 11 years with a sample size of 30. By further restricting to pediatric only, i.e. age 21 or younger, the median age is reduced to 5 years with a sample size of 19 patients. With these criteria, we encountered 9 sIgM^pos^ (47%), and 10 sIgM^neg^ (53%), with low-intermediate expression (scores of “1” to “2” exclusive) included as sIgM^neg^. These results are similar to our original subset of high-FISH-MYC patients, showing that these two subtypes are also discernible in exclusively pediatric patient populations.

**Figure 4:**
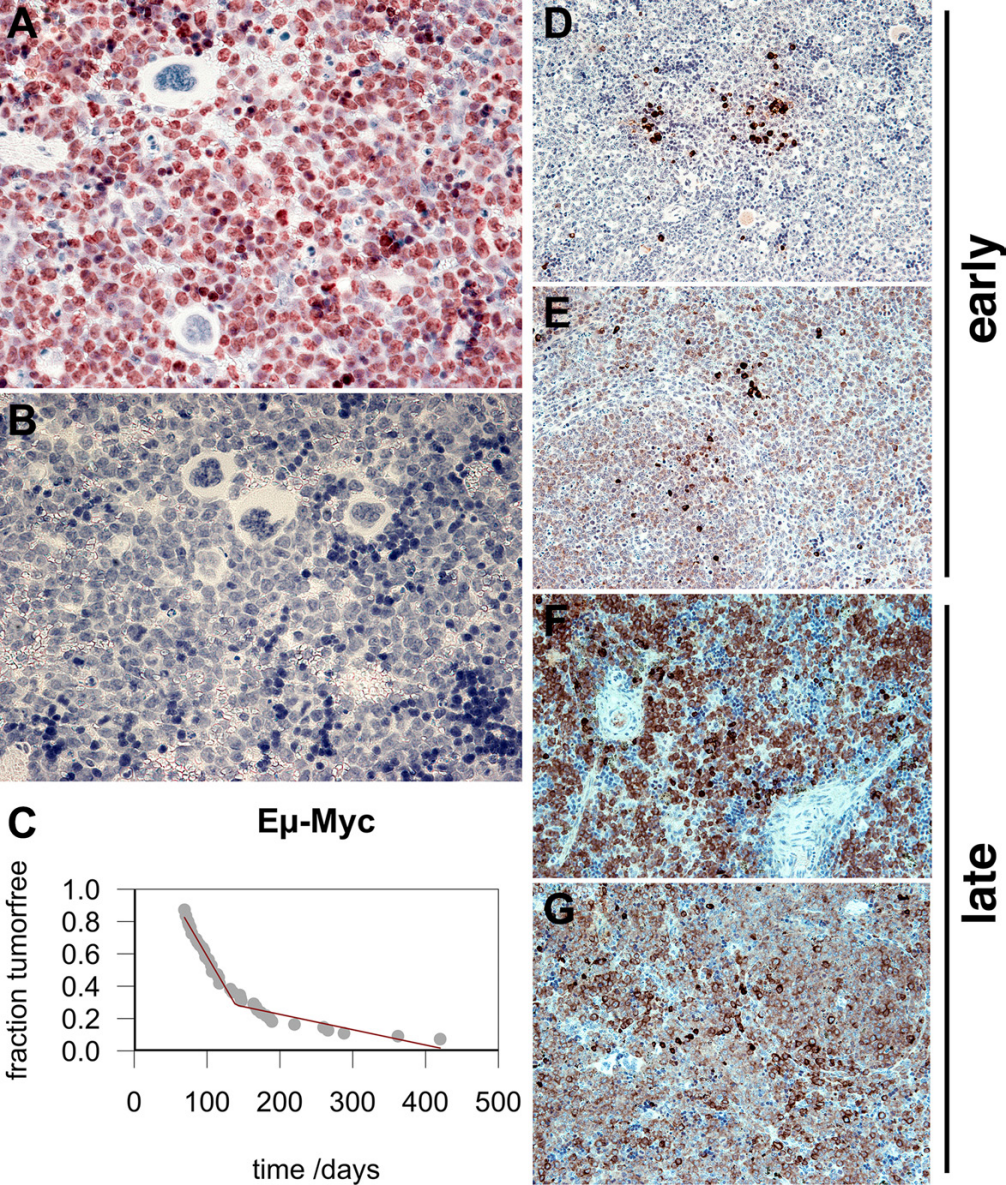
Biphasic tumor incidence and IgM expression of “early” and “late” onset BL in Eμ-Myc mouse model (**A**) Ki-67 stain shown at 400X magnification. (**B**) Reference stain with no primary antibody. Hematoxylin-counterstained nuclei are in blue. Image at 400X magnification. (**C**) Fraction of tumor-free mice at time after birth in the colony. Each gray dot represents one animal. The red line indicates fit by broken stick regression. Also shown are examples of early onset (**D**, **E**) and late onset (**F**, **G**) lymphomas in the Eμ-Myc mouse. IgM staining is dark and concentrated either intracellularly (ic) or on the surface (s). Hematoxylin-counterstained nuclei are in blue. (D, E, F, G) Images at 200× magnification.

**Figure 5:**
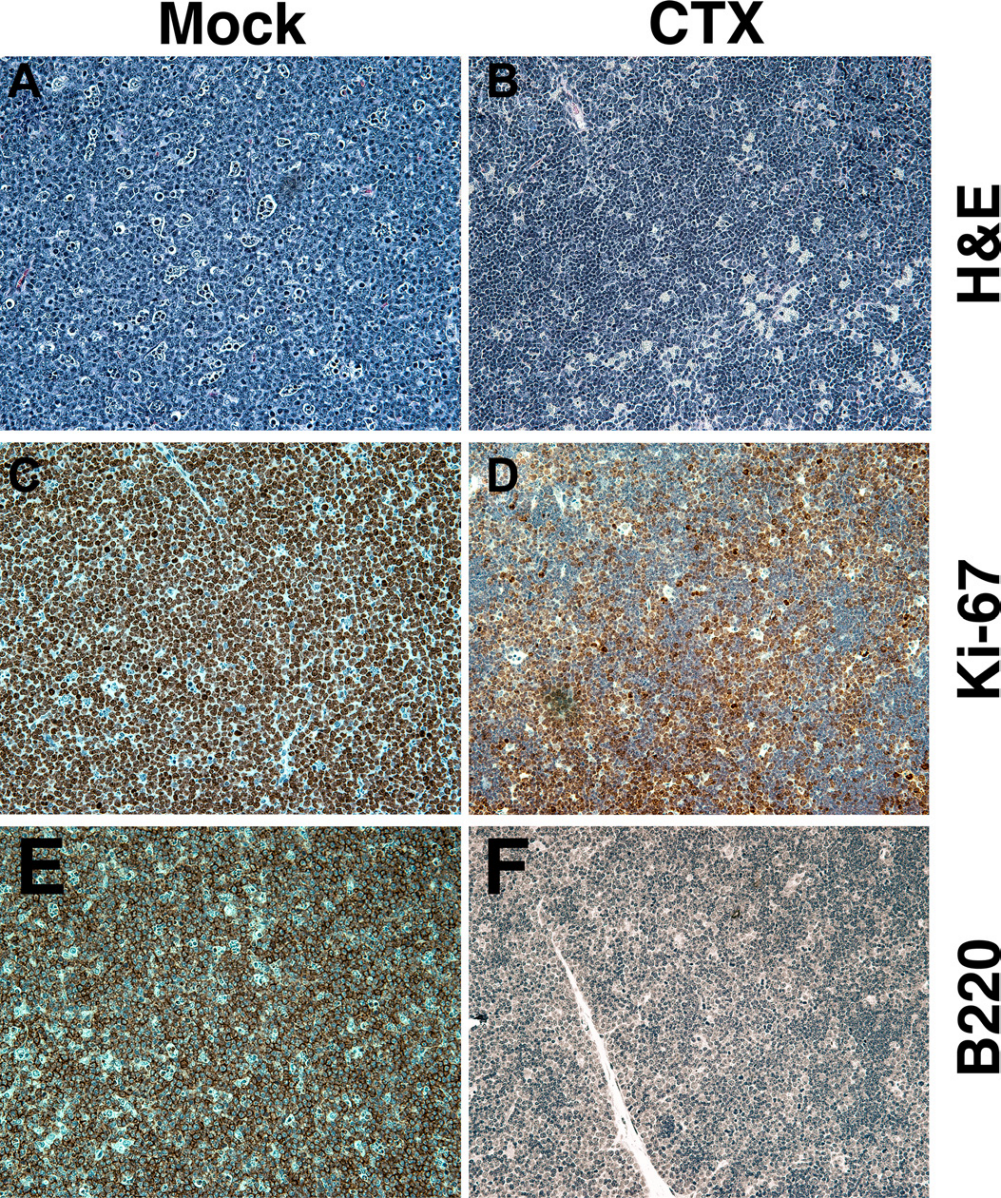
Response to cyclophosphamide in the Eμ-Myc mouse Mock-treated (**A**, **C**, **E**) or cyclophosphamide (CTX) treated BL (**B**, **D**, **F**) in an Eμ-Myc mouse. (A, B) H&E staining. (C, D) Ki-67 staining. (E, F) Murine B220 staining (analogous to human CD19). Hematoxylin-counterstained nuclei are in blue. All images at 200X magnification.

Third, we evaluated sIgM expression in a classic mouse model of BL. In 1985, Adams and Cory developed the first Myc-driven model for BL in mice [12, 13]. This model has been a workhorse for understanding the molecular biology of BL and interventions against this disease ever since. Eμ-Myc mice are immunocompetent and cooperate with Eμ-BCL2 mice in a model of double-hit lymphoma [18]. Eμ-Myc mice develop lymphoma as early as 2 months of age, and the majority dies within 6 months. The initial studies reported a biphasic tumor incidence curve; this has been amply documented since, and we recapitulated this phenotype in our colony (Figure 4, panel C). The tumors exhibit the typical BL “starry-sky” appearance due to the presence of macrophages laden with phagocytized apoptotic cells, and > 95% of the cells stain positive for Ki-67 (Figure 4, panel A). At present, no one molecular difference between “early” and “late” onset lymphoma in the Eμ-Myc model has been reported. Therefore, we tested the hypothesis that sIgM expression differs between “early” and “late” onset lymphoma. Indeed, in the “early” lymphomas, IgM expression was absent except for isolated cells with intracellular (ic) IgM accumulation (Figure 4, panels D and E). By contrast, late onset tumors exhibited universal IgM positivity, the majority of which was surface associated (Figure 4, panels F and G). The tumors responded to single agent cyclophosphamide/Cytoxan (Figure 5, panels B, D, F), which reduced the proliferating B220-positive B cells. This allowed for the non-lymphoma T cells in the mouse spleen to begin to fill in the empty space. We conclude that sIgM positivity also distinguishes two subtypes of lymphoma in the Myc-driven mouse model of BL.

## DISCUSSION

BL is a disease of multiple clinical presentations. The lymphoma originally discovered by Denis Burkitt is a disease clustered in development (early age) and by geography (endemic regions in Africa and Latin America) (reviewed in [1]). It is driven by the t(8;14) translocation, associated with EBV, and has been termed endemic BL (eBL). eBL responds readily to cytotoxic treatment [19]. By contrast, sporadic BL (sBL) is a disease that includes older individuals as well as children, has no geographic preference and a varying degree of EBV association [2]. It is rare in adults by comparison to DLBCL, except in the context of HIV-induced immunodeficiency [6]. This biphasic age distribution and the signature histology of BL are recapitulated in the Eμ-Myc mouse model [12–14, 18].

Molecular profiling studies have established a common signature for sBL that distinguishes it from other types of Myc-translocated and Myc-activated DLBCL [20–22], and have also provided evidence for subtypes within sBL, e.g. those with or without activating mutations in ID3. ID3 mutations were absent in eBL compared to sBL [21, 23, 24]. Thus far, “molecular sBL” signatures were primarily derived from sBL collected in Northern European or US locations and have not distinguished between pediatric or adult patients [25]. To arrive at the molecular expression signatures, sBL was defined as CD20+, BCL6+, CD10+, BCL2-, CD5-, Ki-67 ≥ 95% and IgH-Myc translocation [15, 16]. In the aforementioned profiling studies, 100% of “molecular sBL” carried a BCL6 translocation; yet, only 88% carried the classical IgH-Myc translocation [20]. The “molecular sBL” category included 36 cases of “atypical” sBL because of a non-prototypical immunophenotype, and ultimately led to a 58-gene signature. Dave et al. arrived at very similar molecular signatures [22], and again, the molecularly defined sBL class contained cases that were inconsistent with classification based on the immunophenotypical definition of BL and of PBL [11, 15, 16, 26]. This report provides evidence that there exist additional subtypes of t(8;14) sBL that can be distinguished on the basis of IgM and CD79a expression.

We investigated a set of pediatric BL, each carrying the t(8;14) translocation. We noted first in a case series of sBL from the Southern United States, and then in a validation set of sBL from Brazil, that about half of the cases stained positive for sIgM, and half did not. Because of the high proportion of these two subtypes, and because all stained equally well for other markers (Ki-67, CD10), it is unlikely that the differential IgM positivity was the result of technical variation. Differential IgM expression also correlated with early and late onset lymphoma in the Eμ-Myc transgenic mouse model of BL. Since all Eμ- Myc mice are genetically identical and BL development is extremely rapid, this suggests two different developmental pathways towards BL. In the first scenario, which is characterized by early onset in the mouse, BL development is not dependent on IgM signaling. In the second scenario, which is characterized by later onset in the mouse, the lymphoma cells express high levels of IgM consistent with a dependency on “tonic” BCR signaling [27]. The discovery of sIgM as a novel marker to subtype pediatric sBL provides a new approach to elucidate the biology of BL.

**Figure 6:**
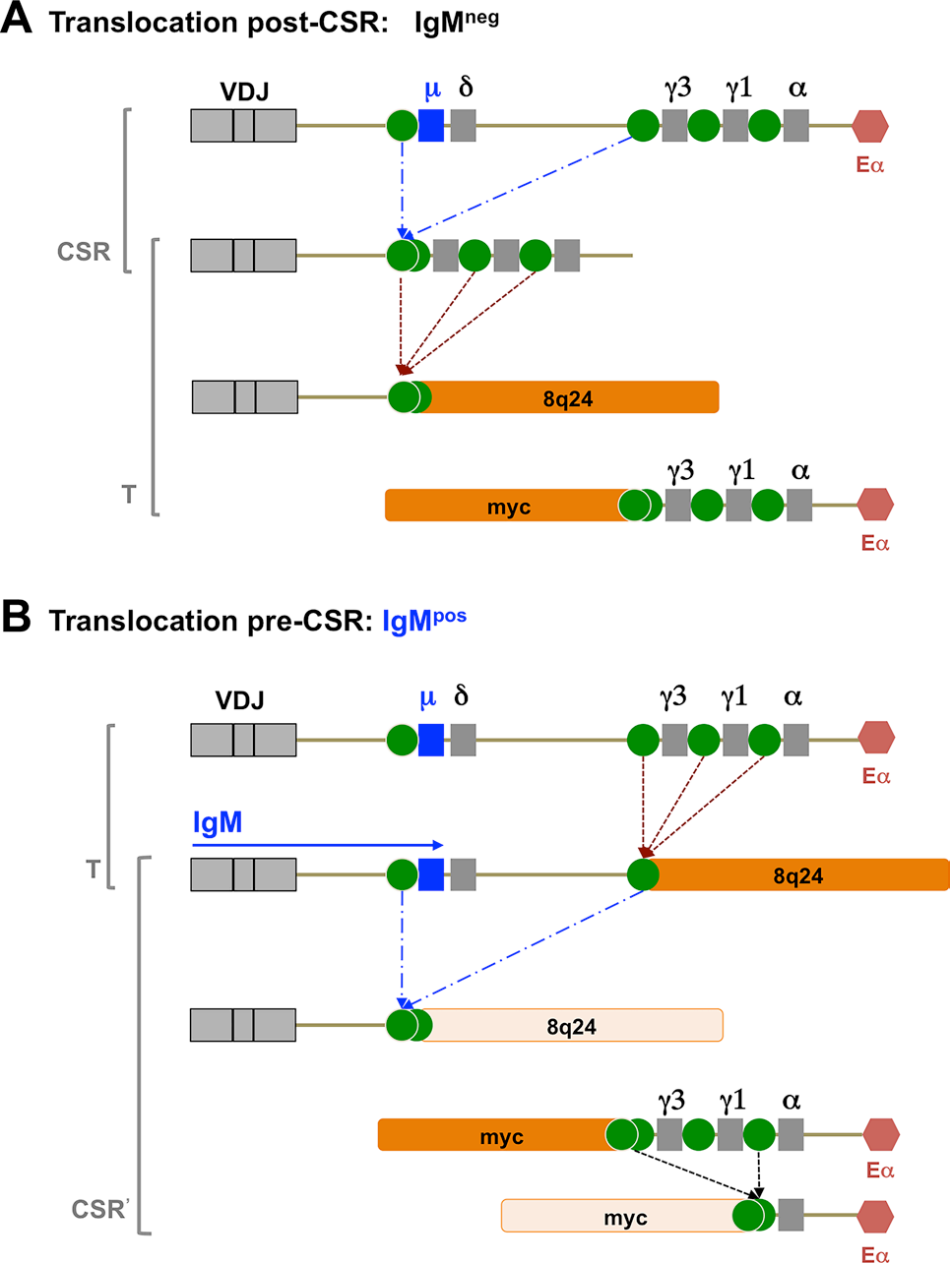
Representative figure of possible CSR events Model of t(8;14) events that can give rise to either sIgM-negative or sIgM-positive BL, depending whether the event takes place post CSR (**A**) or pre-CSR (**B**).

IgM expression can be interpreted in the context of the t(8;14) translocation and class switch recombination (CSR). The t(8;14) translocation juxtaposes Myc to the Eα enhancer, and 8q24 is fused to one of the switch regions (Figure 6, panel A). If CSR takes place before the translocation event, IgM expression would have ceased already as the Cμ exon was replaced by any of the Cγ/ Cα regions. During the translocation 8q24 replaces Cγ/Cα and no isotype is ever expressed. This would correspond to IgM^neg^ BL. If CSR takes place after the translocation event, and if 8q24 is fused to one of the Cγ/Cα switch regions rather than the Cμ switch region, IgM expression would be retained (Figure 6, panel B). This would result in a very broad distribution of translocation points, which has been observed [28]. In either case, the Myc translocation to the 3′ region and to Eα is identical. Thus, IgM^pos^ and IgM^neg^ t(8:14) BL give the same result in the Myc FISH assay. Indeed, Figure 6 is an oversimplification of the human heavy chain locus. Whole genome sequencing established (i) a large duplication of the Cγ/Cα region and (ii) a high frequency of polymorphisms made up of deletions in the human population [29]. This indicates genome instability, in addition to and irrespective of CSR events. If the Myc translocation event took place before CSR, it is at least theoretically possible that CSR can take place afterwards. This event is depicted as CSR' in Figure 6, panel B. The result would again be an IgM-negative t(8;14) BL. There also exists the possibility that CSR takes place on the Myc-translocated fragment, which would bring Myc into even closer proximity to Eα [30]. Another important marker of the pre- or post- CSR origin of BL is somatic hypermutation. Presumably, somatic hypermutation takes place in the cell, cycling back and forth between the light zone and dark zone, and it is dependent on sIg expression. There is conclusive evidence for the presence of somatic hypermutation in IgM^pos^ BL; however particularly in reports on eBL, the extent seems highly variable [31]%. This suggests that the t(8;14) translocation event can occur either before CSR or after CSR.

In summary, IgM expression can distinguish between t(8;14) BL that originate before CSR in the GC (IgM^pos^) and those that originate later in B cell development, after CSR (IgM^neg^); or instances where the t(8;14) tumor cells were nevertheless competent to complete CSR. This establishes IgM as an additional tool to help identify the cell of origin of BL. We observed that IgM expression was paralleled by CD79a expression. CD79a has acquired point mutations in the ITAM motif for a significant (~ 20%) fraction of DLBCL, but none have been reported in BL [32]. The role of BCR signaling in lymphomagenesis is not trivial, since it can transmit activating “tonic,” or “chronic” signals, as well as inhibitory signals in response to Fcγ-RIII [27]. Since CD79a is a requisite mediator of BCR surface translocation and signaling [17], one can conclude that the IgM^neg^CD79a^neg^ BL cells do not depend on positive, nor respond to inhibitory, BCR signaling events. One may speculate that converse is true as well, i.e. IgM^pos^CD79a^pos^ BL still depend on BCR signaling via CD79a as well as its downstream target Bruton's tyrosine kinase (BTK). If so, adding BTK inhibitors like ibrutinib to the treatment regimen may increase efficacy in IgM^pos^CD79a^pos^ BL.

## MATERIALS AND METHODS

### Case selection at UNC chapel hill

Cases were collected at UNC Cancer Hospital and characterized as BL by histology [15, 16]; in short: the tumors showed cohesive growth of monotonous, intermediate-sized abnormal lymphoid cells with immunohistochemical expression of CD10 and CD20, and a high Ki-67 index (> 95%). Tdt and Bcl-2 were negative by immunohistochemistry. BCL-6 data was only available for one patient, which was positive. t(8;14) Myc rearrangement was ascertained by FISH studies. IgM stain intensity was quantified on a scale of 0–3, with 0 as negative, 1 as weak staining, 2 as intermediate, and 3 as strong staining. Intensity score references were created beforehand to ensure consistency in scoring. In the case of large tissue sections, the entirety of the tumor area was evaluated to arrive at a consensus score, and images were taken at varying magnifications of a field representative of the consensus score.

### Brazilian BL TMA

The characteristics of the BL cases were previously described [7]. Each individual case was represented by 3 tumor cores of 0.6 mm diameter, which were obtained via biopsy. Cases were scored by staining intensity on a scale of 0–3, and the 3 tumor scores for each patient were averaged. If the patient only had two or one cores present on the slide due to tissue detachment, the cores present were used for scoring. The majority of cases were male (74%) and under the age of 20 (mean 19.1 years). In each case, the original block was classified as BL according to the 2001 WHO classification. All cases had an immunophenotype of CD20+, CD10+ and Ki-67 > 95%. TdT and Bcl-2 were negative in all cases. ISH for EBV was positive in 52% of cases. In each of these cases, all or virtually all of the neoplastic cells stained positive for EBER1. All cases of the TMA were studied by FISH using the LSI MYC Dual-Color Break apart Rearrangement probe.

### Eμ-Myc mice

Eμ-Myc mice (*n* = 51) were obtained from Jackson laboratory (strain C57BL/6J-Tg(IghMyc)22Bri/J) and bred at UNC-Chapel Hill. All animals were heterozygous for the Eμ-Myc transgene. Animals were followed visually and palpated every two days. The tumors were excised upon necropsy and fixed in 5% formalin/PBS (Fisher Diagnostics, Middletown, VA). All animal work was conducted according to AAALAC guidelines and was approved by the UNC IACUC committee.

### Immunohistochemistry

Formalin-fixed, paraffin embedded BL tumors from the University of North Carolina were cut into 7 μm thick sections and mounted onto glass slides. Slides were dried at 58°C for 3 hours and then placed into a Dako PT Link Module (Dako North America Inc, CA, USA) for pretreatment at 75°C in 10 mM, pH 6.0 sodium citrate buffer, supplemented with 0.05% Tween-20. Buffer temperature was raised to 96°C for 25 minutes, and then lowered to 75°C. Slides were deparaffinized in room temperature diluted wash buffer (Dako North America Inc, CA, USA). Endogenous peroxidase activity was blocked with 3% hydrogen peroxide. A series of PBS washes was done between all subsequent steps. Samples were blocked for 30 minutes with 1.5% animal serum from VectaStain™ Elite ABC Kits (Vector Laboratories, CA, USA) diluted in PBS and supplemented with 1% bovine serum albumin, 0.1% cold water fish skin gelatin, 0.1% Triton X-100, 0.05% Tween-20 and 10% Avidin from an Avidin/Biotin Blocking Kit (Vector Laboratories, CA, USA). Sections were incubated overnight at 4°C in either the antibody diluent containing 10% biotin, 1% bovine serum albumin, 0.1% cold water fish skin gelatin, and 0.1% Triton X-100, or a dilution of the primary antibody in the same diluent. The appropriate diluted secondary antibody supplemented from the respective ABC kit was applied to the sections for 30 minutes. Sections were incubated in the ABC reagent for 30 minutes, and then developed with Vector NovaRed™ substrate for 5 minutes and counterstained with 0.1% Mayer's Hematoxylin (Electron Microscopy Sciences, PA, USA). They were dehydrated and cleared by a series of 5-minute washes in ethanol and Histochoice™ Clearing Agent (Amresco Inc, OH, USA), which was automated using a Linear Stainer instrument (Leica, Heidelberg, Germany). Slides were mounted using Cytoseal™ (Richard-Allan Sci., MI, USA) and glass coverslips.

### Primary antibodies

Ki-67 antibody was used at a 1:100 dilution (Thermo Lab Vision, CA, USA), human IgM at 1:400 (Novocastra Lab Ltd, Newcastle, UK), and CD79a at 1:100 (Novocastra Lab Ltd, Newcastle, UK). Sections were imaged using a Leica DM LA histology microscope (Leica, Heidelberg, Germany) equipped with a 20/0.70 numerical aperture (NA) or a 40/0.75 NA N plan objective and Leica DPC480 camera. Images were stored as TIFF files under Mac OS X10.8.5.
